# The amniotic fluid proteome changes across gestation in humans and rhesus macaques

**DOI:** 10.1038/s41598-023-44125-3

**Published:** 2023-10-09

**Authors:** Lyndsey E. Shorey-Kendrick, B. Adam Crosland, Eliot R. Spindel, Cindy T. McEvoy, Phillip A. Wilmarth, Ashok P. Reddy, Keith D. Zientek, Victoria H. J. Roberts, Rahul J. D’Mello, Kimberly S. Ryan, Amy F. Olyaei, Olivia L. Hagen, Matthew G. Drake, Owen J.T. McCarty, Brian P. Scottoline, Jamie O. Lo

**Affiliations:** 1 Division of Neuroscience, Oregon National Primate Research Center, Oregon Health & Science University, Beaverton, OR USA; 2 Division of Maternal-Fetal Medicine, Department of Obstetrics and Gynecology, Oregon Health & Science University, Portland, OR 97239 USA; 3 Division of Neonatology. Department of Pediatrics, Oregon Health & Science University, Portland, OR USA; 4 Proteomics Shared Resources, Oregon Health & Science University, Portland, OR USA; 5 Division of Reproductive & Developmental Sciences, Oregon National Primate Research Center, Oregon Health & Science University, Beaverton, OR USA; 6 Division of Pulmonary and Critical Care Medicine, Oregon Health & Science University, Portland, OR USA; 7 Department of Biomedical Engineering, Oregon Health & Science University, Portland, OR USA

**Keywords:** Translational research, Evolutionary developmental biology, Rhesus macaque

## Abstract

Amniotic fluid is a complex biological medium that offers protection to the fetus and plays a key role in normal fetal nutrition, organogenesis, and potentially fetal programming. Amniotic fluid is also critically involved in longitudinally shaping the in utero milieu during pregnancy. Yet, the molecular mechanism(s) of action by which amniotic fluid regulates fetal development is ill-defined partly due to an incomplete understanding of the evolving composition of the amniotic fluid proteome. Prior research consisting of cross-sectional studies suggests that the amniotic fluid proteome changes as pregnancy advances, yet longitudinal alterations have not been confirmed because repeated sampling is prohibitive in humans. We therefore performed serial amniocenteses at early, mid, and late gestational time-points within the same pregnancies in a rhesus macaque model. Longitudinally-collected rhesus amniotic fluid samples were paired with gestational-age matched cross-sectional human samples. Utilizing LC–MS/MS isobaric labeling quantitative proteomics, we demonstrate considerable cross-species similarity between the amniotic fluid proteomes and large scale gestational-age associated changes in protein content throughout pregnancy. This is the first study to compare human and rhesus amniotic fluid proteomic profiles across gestation and establishes a reference amniotic fluid proteome. The non-human primate model holds promise as a translational platform for amniotic fluid studies.

## Introduction

The in utero environment plays a significant role in fetal development and is vulnerable to adverse maternal environmental factors including chronic stress, environmental exposure, substance use, infection, and metabolic disease. A critical entity that influences fetal development in utero is amniotic fluid^1,2^. It is a complex biological medium that surrounds the fetus, offers mechanical protection, provides nutrition, is critical for normal fetal growth and organogenesis, and can potentially influence fetal programming^3–6^. Amniotic fluid is swallowed by the fetus as early as 16 weeks of human gestation and plays a vital role in the development and maturation of the fetal gastrointestinal tract, lungs, and immune system^1,4,6–9^. In vitro studies have shown that amniotic fluid has intestinal epithelial cell trophic effects equivalent to breast milk and prior research has demonstrated the benefit of simulated amniotic fluid in reducing feeding intolerance in preterm human infants^10^.

Despite its importance, the biology of amniotic fluid has been understudied. The existing literature suggests that the composition of amniotic fluid is dynamic and changes across gestation^11,12^. It is rich in proteins, lipids, oligosaccharides, ribonucleic acids (RNAs), and metabolites that potentially influence fetal development through bioactivities^12^. Amniotic fluid proteins and metabolites from human cross-sectional samples appear to evolve over the course of pregnancy^13,14^. However, alterations between different trimesters have not been confirmed within the same pregnancy because serial amniotic fluid sampling is not typically performed in human pregnancies.

The knowledge deficit regarding the biology of amniotic fluid is partly because of the challenges in obtaining amniotic fluid samples from healthy human pregnancies, but also because of the limited translational strength of most animal models for amniotic fluid research. Human amniotic fluid studies typically examine samples collected at 16–20 weeks of gestation when amniocentesis is clinically performed for genetic or infectious testing. In addition, usually only one sample of amniotic fluid is collected before viability (approximately less than 23 weeks gestation) during a human pregnancy. This limits studies to a cross-sectional design, which does not allow insight into the potential longitudinal evolution of amniotic fluid composition within individual pregnancies. Consequently, detailed understanding of how amniotic fluid composition changes over gestation is limited, as is our understanding of the influence of the maternal environment on amniotic fluid composition, including the proteome^15,16^.

Non-human primate (NHP) models are an ideal proxy for studying human amniotic fluid biology because of their similar genome and developmental ontogeny. In addition, NHP models allows a high degree of experimental and environmental control and the ability to obtain serially collected amniotic fluid samples from the same pregnancy, which is not ethical or feasible to perform in humans. To address this research gap and overcome these obstacles, we employed a rhesus macaque NHP model to collect longitudinal amniotic fluid samples within the same healthy pregnancies at three time points across gestation. Tandem mass tag (TMT) isobaric labeling quantitative proteomic analyses were then performed with gestational age-matched human amniotic fluid samples collected cross-sectionally. Our objectives were to determine the translational strength of the rhesus macaque model for human amniotic fluid studies and to establish a reference proteomic profile across gestation to identify adversely affected pregnancies in future work. This is an important first step that will establish a pipeline for future studies and ultimately provide insights into the role of amniotic fluid protein composition on fetal development and the in utero environment, which define later-life offspring outcomes.

## Results

### Demographic characteristics of study population

Characteristics of the subjects that served as the source of amniotic fluid are shown in Table 1 for each species. The rhesus macaque samples were obtained from 7 healthy pregnant animals (resulting in 2 male and 5 female offspring) with an average maternal age of 9.1 years (Range: 7–13 years) and median parity of 3. Maternal weights averaged 8.5 kg and varied minimally across subjects. Human samples were obtained from 24 subjects (3 samples were from a de-identified sample bank and demographic data was not available). Maternal age of the human subjects averaged 32.6 years (Range: 18–41), had an average weight of 79 kg (Range: 61.8–129.1 kg) at the time of amniocentesis, and 57% (12/21) of the fetuses were female. The majority of early human samples were obtained for routine genetic testing and were normal at birth; additional details including indications for amniocentesis in human subjects are provided in Supplementary Table 1 (Table S1).

**Table 1:** Demographics and sample characteristics by species and gestational age.

Characteristic	Rhesus	Human
Total number of subjects	7 (measured longitudinally)	24 (21)^#^
Maternal age (years)	9.14 ± 2.12	32.6 ± 5.19
Parity	3 ± 0	0 ± 0
Maternal weight (kg)	8.51 ± 0.92	n.m
Fetal birth weight (g)	487.72 ± 101.85 (3)	2876.72 ± 573.49 (18)
Fetal sex	2 male, 5 female	9 male, 12 female

Continuous variables are expressed as mean ± SD and categorical variables are expressed as median ± IQR.

^#^Three human amniotic fluid samples were obtained from a de-identified bank and are not included in any summaries of patient demographics; (n) indicates a number of samples with information if incomplete; n.m. = not measured in any samples.

### Rhesus macaque and human amniotic fluid proteins share high homology

**Figure 1:**
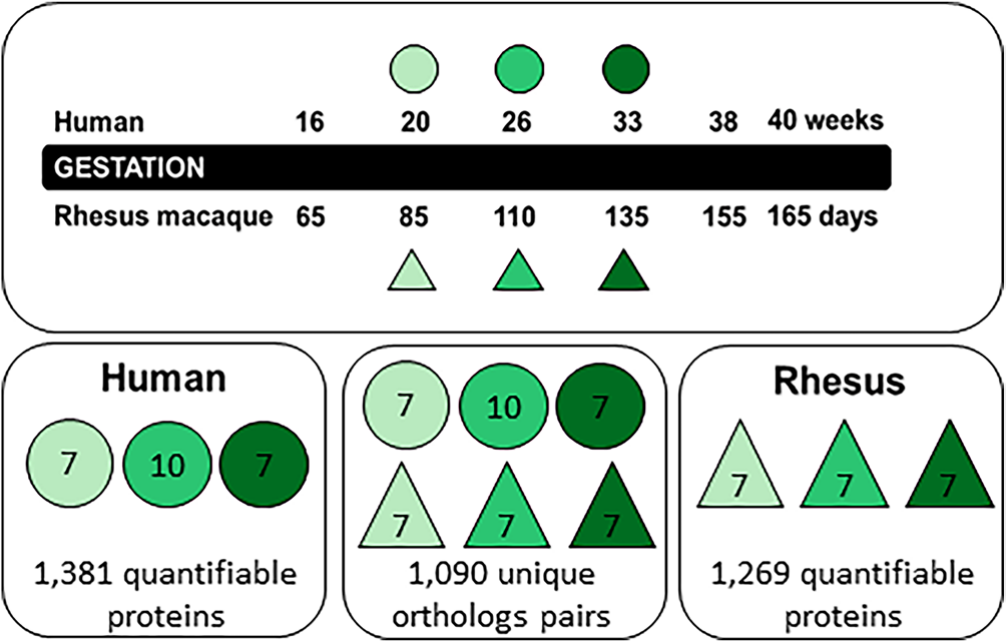
Study design and summary of quantifiable proteins. The rhesus macaque amniotic fluid samples were collected from the same animals at 3 gestational ages (in days): G85, G110, and G135 (21 samples total). These gestational time points were matched by percent completed gestation with banked human samples (24 samples total). The comparable human gestational age time point for G85 is approximately 20 weeks (sample range 19 5/7 weeks to 20 4/7 weeks), for G110 approximately 26 weeks (sample range 24 1/7 to 26 2/7 weeks), and for G135, approximately 33 weeks (sample range 30 3/7 to 31 3/7 weeks). Samples from rhesus and human were isobarically labeled and pooled for proteomic analysis. We determined the relative abundances of 1381 proteins in human samples, 1269 in rhesus samples, and 1090 unique homologs shared across species.

**Figure 2:**
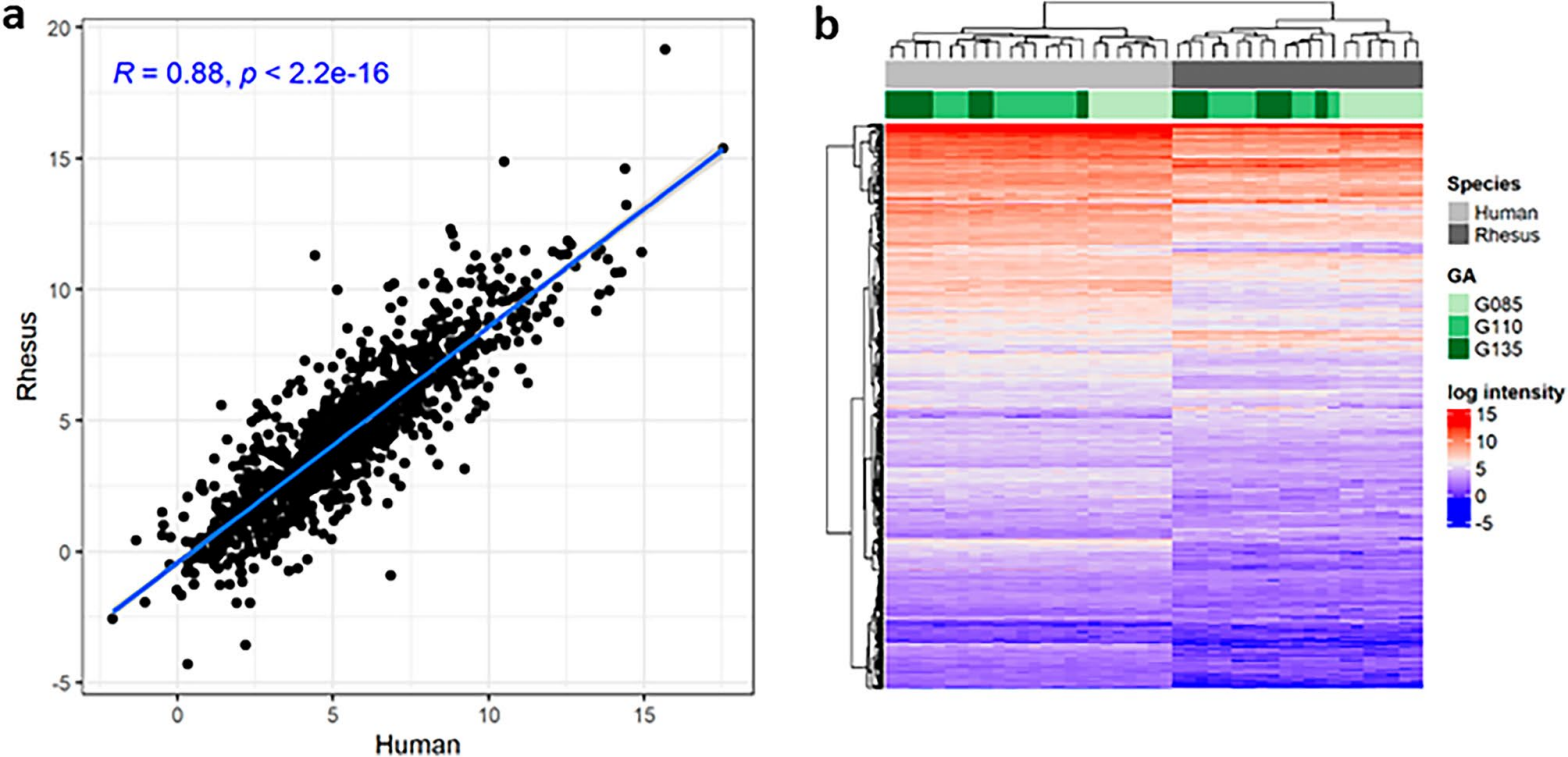
Rhesus and human amniotic fluid proteins are highly homologous. In the 1090 protein ortholog pairs shared between human and rhesus amniotic fluid, the abundance (log intensity) was highly correlated across species at rhesus equivalent gestational age windows (**a**). Hierarchical clustering of all samples and ortholog pairs revealed clear separation of samples by species and clustering of samples by rhesus equivalent gestational age within a species (**b**).

In this study, 24 human and 21 rhesus amniotic fluid samples were multiplexed using isobaric labeling and randomly distributed across 3 separate multi-dimensional LC–MS/MS runs. Common sample pools in all 3 plexes were used to perform internal reference scaling so that reporter ion intensities from all 45 samples were on a common protein abundance measurement scale. Species-specific FASTA searches were performed in parallel and yielded 1269 quantifiable proteins from rhesus amniotic fluid samples and 1381 quantifiable proteins in human amniotic fluid samples. The median inter-sample coefficient of variation was 24.4% in human and 29.8% in rhesus. After removing immunoglobulin family proteins, there were 1204 quantifiable rhesus proteins, of which 1148 (95.5%) overlapped with human proteins (Fig. 1). We reduced this protein set further to 1090 uniquely mapping orthologs for comparison of protein abundance across species. Within these unique orthologs, there was a significant correlation of mean protein intensities across species (R = 0.88; p < 2.2e–16; Fig. 2a), although hierarchical clustering of log protein intensity still separated samples by species (Fig. 2b). The correlation (R values) between species was similar across all rhesus equivalent ages (G85 = 0.89, G110 = 0.87, and G135 = 0.86). The most abundant protein in rhesus amniotic fluid was IGFBP1, which was the third most abundant protein in human amniotic fluid. Among the top 100 most abundant proteins in either species, 70 overlapped including several complement component proteins, glycoproteins, and growth factor proteins (Table S2).

### Amniotic fluid proteome composition changes significantly with gestational age

**Figure 3:**
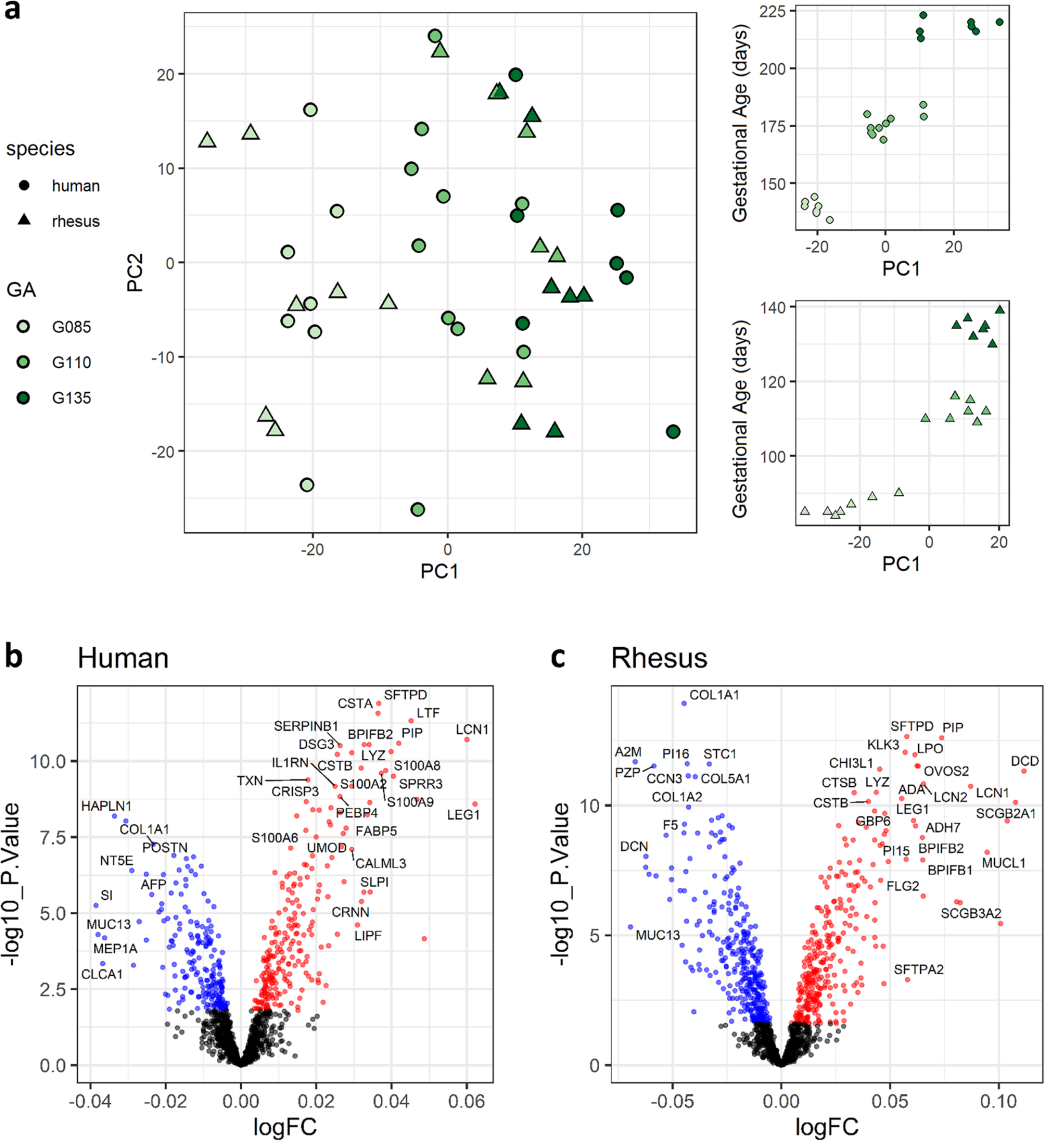
Gestational age strongly affects the amniotic fluid proteome. Principal component analysis was performed for human and rhesus datasets separately and then PC1 was plotted against PC2 for both species together, and against rhesus equivalent gestational age windows for each species separately (**a**). For human amniotic fluid (**b**) and rhesus (**c**), the differential abundances associated with longitudinal gestational age (measured in actual days) are shown in volcano plots with the log2 transformed fold change on the x-axis and the – log10Pvalue on the y-axis. Red indicates increased abundance with gestational age, and blue is decreased.

**Figure 4:**
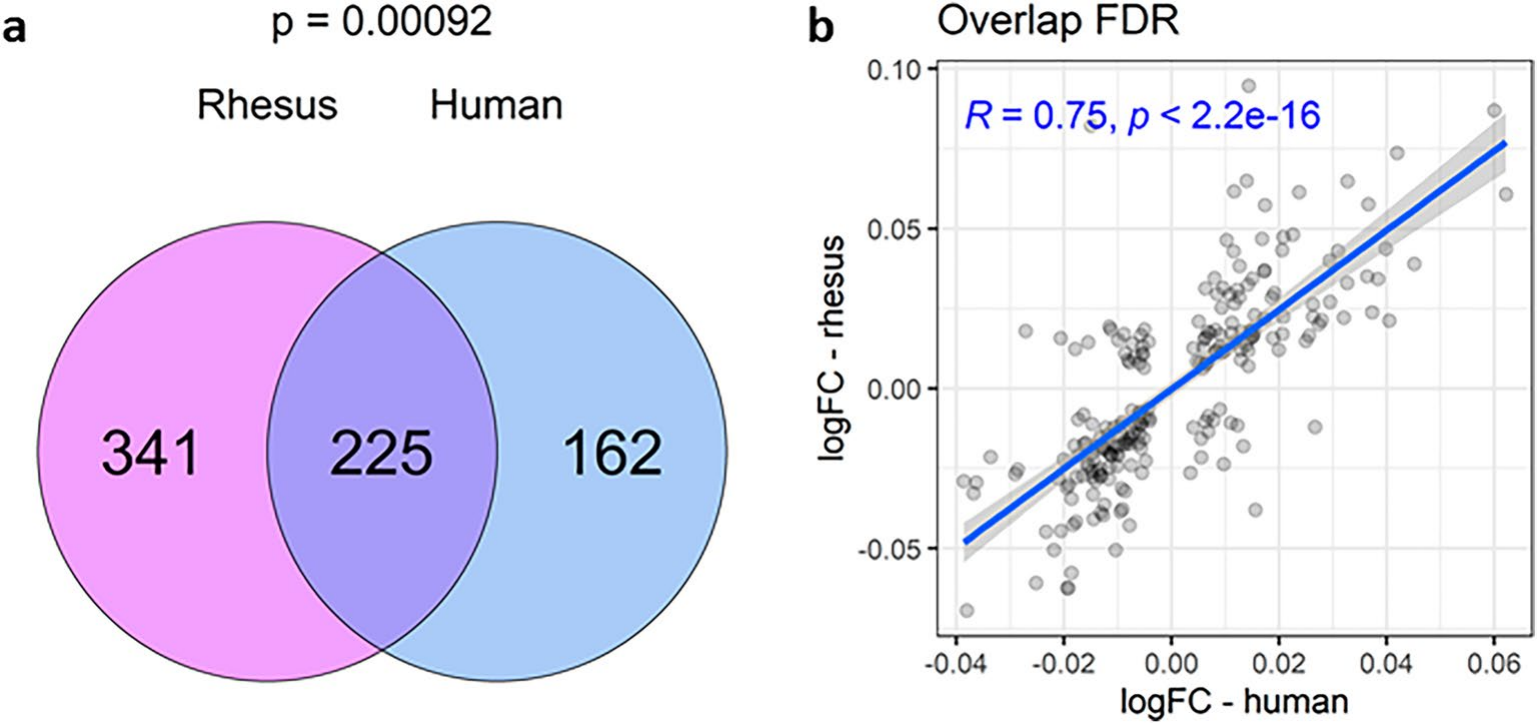
Cross-species comparisons of gestational age-dependent changes in amniotic fluid proteome. The Venn diagram depicts the overlap of gestational-age-associated proteins in amniotic fluid between species (**a**) at FDR significance and the p-value indicates an enrichment of overlap using the hypergeometric test. The scatterplot (**b**) shows the effect size (log fold change) of gestational age for these overlapping proteins with effect size in human samples on the x-axis relative to the effect size in rhesus samples on the y-axis. The blue line and text indicates the Pearson correlation, with the 95% confidence interval shown in grey.

Principal component analysis of the amniotic fluid proteome in either human or rhesus revealed clear separation of samples by gestational age. The first principal component (PC1) explained 21% and 27% of variability in human and rhesus datasets, respectively, and PC1 was strongly associated with rhesus equivalent gestational age within each dataset (Fig. 3a). Out of 1381 proteins measured in human amniotic fluid, a total of 440 proteins (31.9%) were differentially expressed with gestational age (238 down; 202 up**; **Fig. 3b). Of the 1269 proteins measured in rhesus amniotic fluid, 638 proteins (50.3%; 308 down; 330 up) were differentially expressed with gestational age (Fig. 3c). The top proteins changing with gestational age over time (based on actual sample age in days) are shown in Table 2, and the full lists of identified proteins and their association with gestational age are provided in Tables S3 and S4. Of the 1090 shared ortholog proteins in both datasets (Table S5), 35% did not change with gestational age in either species, 17.2% were associated with gestational age in both species in the same direction (188 proteins; 89 up; 99 down), 44.3% were significantly associated with gestational age in only one species, and only 3.4% (37 proteins) were significantly associated with gestational age in opposite directions. Out of 387 proteins associated with gestational age in human amniotic fluid, 225 (58%) were also associated with gestational age in rhesus and the correlation of effect sizes between the two species in the 225 overlapping false discovery rate (FDR) significant proteins was highly significant (R = 0.75; p < 2.2e–16; Fig. 4).

**Table 2:** Top differentially expressed proteins with gestational age.

	Identifier	UniProt GeneName^#^	logFC	P.value	Adj. Pval
Rhesus	F7BJV7_MACMU	COL1A1	− 0.04	1.18E-14	1.49E-11
	SFTPD_MACMU	**SFTPD**	0.06	2.21E-13	1.05E-10
	A0A5F7ZLR3_MACMU	**PIP**	0.07	2.49E-13	1.05E-10
	KLK3_MACMU	KLK3	0.06	8.84E-13	2.81E-10
	F7BAC5_MACMU	LPO	0.06	1.11E-12	2.83E-10
	A0A5F7Z9H8_MACMU	A2M	− 0.07	2.07E-12	3.53E-10
	F6RSK7_MACMU	PI16	− 0.04	2.47E-12	3.53E-10
	A0A5F8ALF6_MACMU	STC1	− 0.03	2.54E-12	3.53E-10
	F7GGG0_MACMU	CA6	0.06	2.96E-12	3.53E-10
	A0A1D5QF45_MACMU	PZP	− 0.06	3.02E-12	3.53E-10
Human	SFTPD_HUMAN	**SFTPD**	0.04	1.28E-12	1.76E-09
	CYTA_HUMAN	CSTA	0.04	2.68E-12	1.85E-09
	TRFL_HUMAN	LTF	0.05	4.74E-12	2.18E-09
	LCN1_HUMAN	LCN1	0.06	1.96E-11	5.32E-09
	PIP_HUMAN	**PIP**	0.04	2.57E-11	5.32E-09
	TCO1_HUMAN	TCN1	0.03	2.85E-11	5.32E-09
	BPIB2_HUMAN	BPIFB2	0.03	2.89E-11	5.32E-09
	ILEU_HUMAN	SERPINB1	0.03	3.08E-11	5.32E-09
	LYSC_HUMAN	LYZ	0.04	4.85E-11	7.30E-09
	CYTB_HUMAN	CSTB	0.03	5.29E-11	7.30E-09

^#^Annotation to human ortholog gene symbol if an acceptable match was made. Proteins common to both species are highlighted in bold.

### Functional enrichment of gestational age-related amniotic fluid proteins is consistent across species

**Figure 5:**
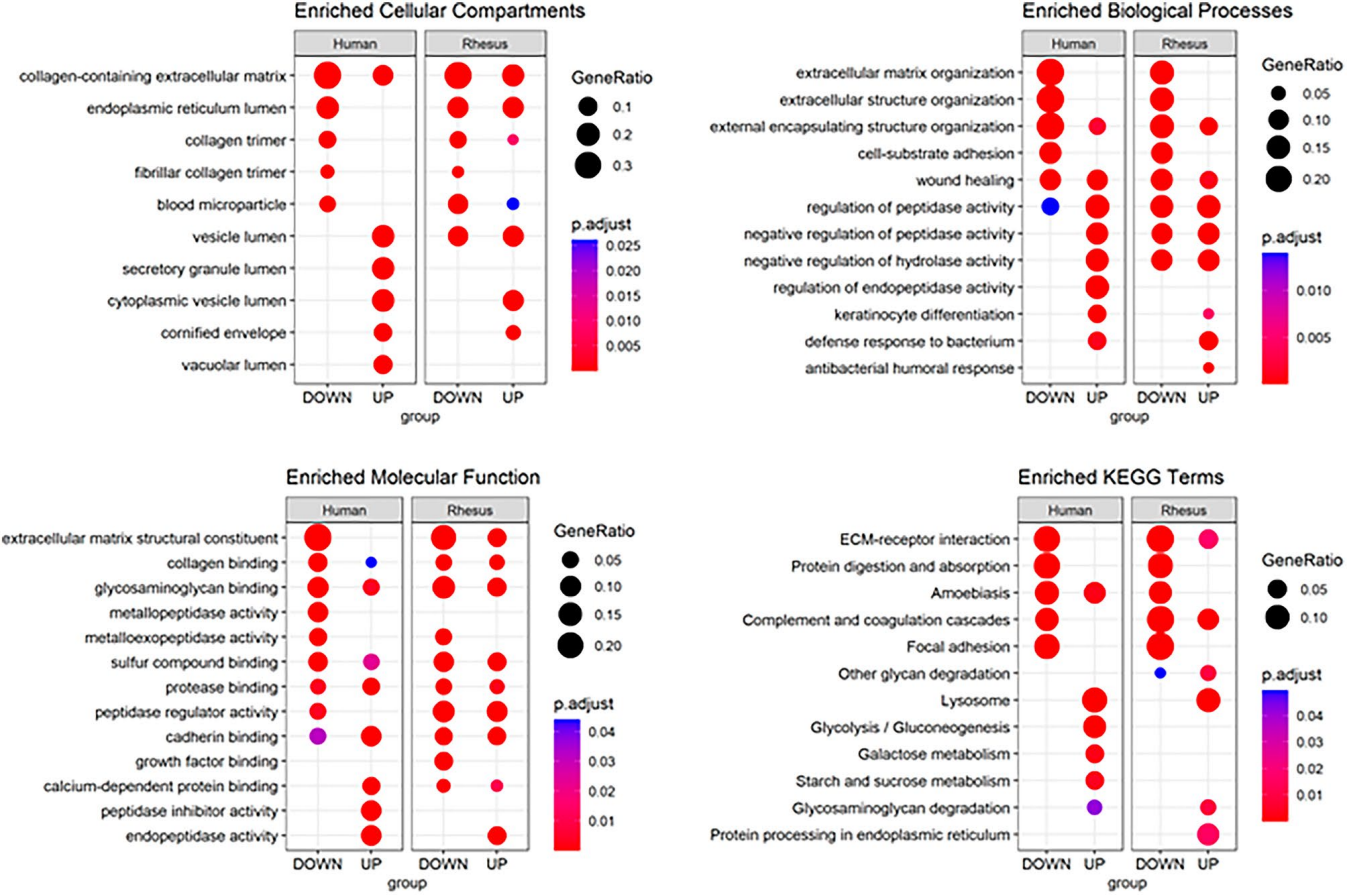
Functional enrichment of amniotic fluid proteins associated with gestational age. Gestational age associated proteins in amniotic fluid are enriched similarly across species. Enrichment analyses were performed using the compareCluster function to identify enriched GO terms and KEGG pathways, separately. Each figure contains two panels (human and rhesus) and separates direction of effect (DOWN/UP) on the x-axis. Enriched categories are indicated on the y-axis. The sizes of points indicate the relative GeneRatio (size of overlap of proteins in our list versus all proteins in the list for that category) and the color indicates the relative p-value of enrichment, with red indicating a lower adjusted p-value.

We next compared functional terms overrepresented among the amniotic fluid proteins associated with gestational age across species and by direction of effect. The top 5 unique terms per category and subgroup are presented in Fig. 5, and the full list of terms is presented in Table S6. The most enriched Cellular Compartment Gene Ontogeny (GO) term for downregulated proteins in both species was “collagen-containing extracellular matrix”. The most enriched GO terms among upregulated proteins were “endoplasmic reticulum lumen” in rhesus and “secretory granule lumen” in human amniotic fluid. Similarly, enriched Biological Process and Molecular Function GO identifiers within downregulated proteins included many terms related to extracellular matrix, wound healing, and coagulation. Overrepresented among upregulated amniotic fluid proteins by gestational age were several terms related to peptidase activity, cellular compartment lumens, and immune response. The top Kyoto Encyclopedia of Genes and Genomes (KEGG) Pathways include “ECM-receptor interaction” and “protein digestion and absorption” among downregulated proteins in both species, while upregulated proteins were enriched for “lysozyme” and “glycosaminoglycan degradation” in both species. Top Canonical Pathways activated in rhesus amniotic fluid proteins changing with gestational age included “LXR/RXR Activation,” “Complement System,” and “GP6 Signaling Pathway,” and in human amniotic fluid proteins the top 3 Canonical Pathways were “GP6 Signaling Pathway,” “Pulmonary Fibrosis Idiopathic Signaling Pathway,” and “Gluconeogenesis I.”

### Enrichment of tissue-specific signature in amniotic fluid proteins associated with gestational age

**Figure 6:**
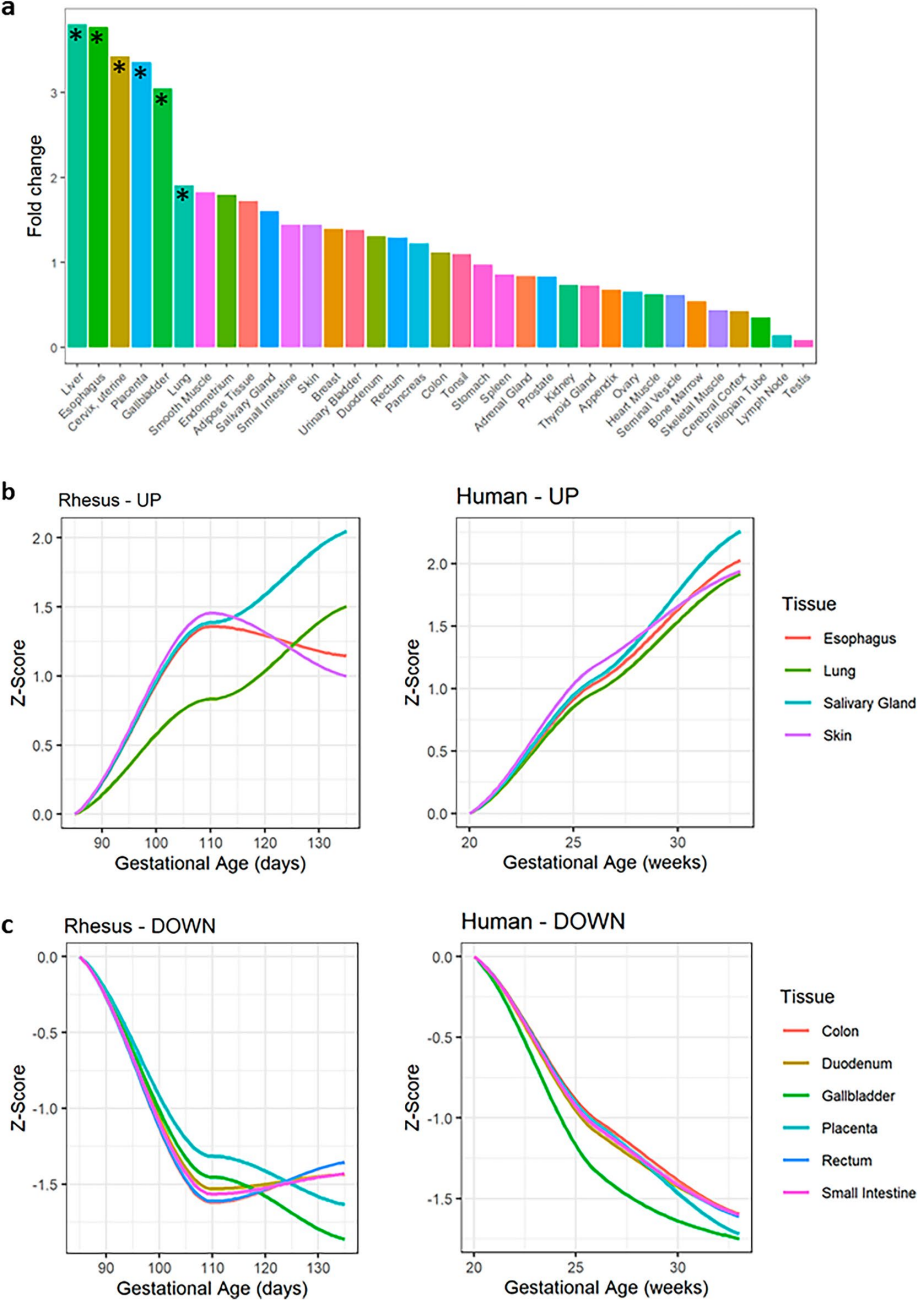
Enrichment and gestational age dependence of tissue-specific signatures in amniotic fluid. Analysis of all quantifiable proteins in amniotic fluid identified enrichment of liver, placenta, esophagus, cervix, gallbladder, and lung signatures (**a**) based on the Human Protein Atlas dataset^17^. Tissue enrichment analysis was performed separately for orthologs increasing or decreasing in both species. For each enriched tissue, we summarized expression of all proteins over-represented in that tissue using a Z-score normalized to abundance at the earliest gestational age. The tissue-specific trajectories over gestational age are shown for upregulated (**b**) and downregulated (**c**) proteins separately for rhesus samples (left) and human samples (right).

To gain further insight into the biological source and function of amniotic fluid proteins modulated by gestational age, we also performed tissue enrichment analysis using the Human Protein Atlas (HPA) as a reference^17^. Analysis of all quantifiable proteins in amniotic fluid identified enrichment of liver, placenta, esophagus, cervix, gallbladder, and lung signatures (Fig. 6a). Among orthologs with increasing expression in both species, the most enriched tissues were esophagus (e.g. SPRR3 and CRNN), skin (e.g. DSP and FLG), lung (e.g. SFPTB, SFPTD, and SCGB1A1), and salivary gland. In humans, the average trajectory of upregulated proteins within each tissue group continued to increase with gestational age, while in rhesus the average abundance of esophagus and skin-enriched proteins plateaued at mid-gestation between gestational days 110 (G110) to G135 (term is ~ 168 days) (Fig. 6b). Unique proteins increasing with gestational age in rhesus amniotic fluid (Fig. S1) were most enriched for cervix (uterine) tissue expression (e.g. SCGB2A1 and SCGB3A1), while unique proteins increasing with gestational age in human amniotic fluid (Fig. S2) were most enriched for esophageal tissue expression (e.g. multiple serpin and kallikrein family members). The most enriched tissues among proteins decreasing with gestational age in both species were placenta, small intestine, gallbladder, colon, cervix, and rectum. Again, in humans the average expression within each tissue-enriched group continued to decrease with gestational age, while in rhesus the average abundance of gastrointestinal-enriched (e.g. rectum, small intestine, and duodenum) proteins plateaued between G110 to G135 (Fig. 6c). Unique to rhesus was the enrichment of liver proteins among downregulated proteins in amniotic fluid such as FETUB (fetuin B), TF (transferrin), and PZP (pregnancy zone protein) (Fig. S1). Proteins decreasing with gestational age in human amniotic fluid only were most enriched for placental tissue expression (e.g. FBN2—placensin) (Fig. S2).

### Gestational age-associated protein changes are consistent with previous studies

We compared our list of human amniotic fluid proteins associated with gestational age with a list of 320 proteins differentially expressed between mid-gestation and term non-labor samples^18^. We identified 165 of the 320 previously observed amniotic fluid proteins in human amniotic fluid samples from our study, and 75 of the 165 were consistently differentially expressed with gestational age (R = 0.86; p < 2.2e–16; Fig. S3). Proteins increasing most with gestational age in either study included LTF, LPO, and LYZ; while HAPLN1, AFP, and DCN decreased the most. Amniotic fluid proteins uniquely associated with gestational age in our study included SFTPD, CSTA, and LCN1 which increased with gestational age, and IGSF1, COL1A1, and FBLN1 which decreased with gestational age.

## Discussion

Amniotic fluid is a complex biological fluid with roles during pregnancy primarily conceived of as supportive and protective, including mechanical protection of the fetus, environmental homeostasis, and supporting lung development through fluid pressure. Less recognition is given to the additional roles of amniotic fluid, such as a source of nutrition, demonstrated by ^35^S-labelled amniotic fluid protein uptake into fetal tissues^19^, as well as the high frequency of fetal growth restriction in the setting of congenital gastrointestinal disorders^1,20–23^. Alterations in amniotic fluid from severe oligohydramnios or anhydramnios have been shown to influence gastrointestinal development^4,23^ and, if occurring prior to or in the second trimester, are strongly associated with pulmonary hypoplasia^24^.

The data presented here, combined with prior studies^18,25,26^, demonstrates that amniotic fluid contains well over a thousand proteins. The amniotic fluid proteome consists of many functional proteins, some of which are relatively specific to amniotic fluid and have not been reported in other biological fluids^27^. Comparison of the available amniotic fluid proteome data is complicated by differing gestational ages at the time of collection, indications for sample collection, methods of sample collection and preparation, and different proteomic and bioinformatic approaches. Despite these limitations, the current data and previous reports^18,28^ suggest that amniotic fluid has a highly complex and potentially unique protein composition.

A barrier to amniotic fluid research has been the lack of a suitable research model for the study of amniotic fluid longitudinally. The study of amniotic fluid using a NHP model offers several advantages over other model systems due to their similarity to humans, including placentation^29,30^, gestational period, developmental ontogeny^31,32^, and genome, with an average of ~ 93% human-rhesus sequence identity^33^. Use of a NHP model also allows experimental control not readily achievable in humans, and the ability to serially sample amniotic fluid at multiple gestational time points from the same pregnancy, which is neither ethical nor feasible in humans. We demonstrate, for the first time, remarkable homology of the human and rhesus amniotic fluid proteomes, with ~ 96% protein identity and strong correlation of gestational age-matched mean intensities (R = 0.88). The coefficient of variation for both rhesus and human amniotic fluid proteomes was small, likely reflecting both the stability of amniotic fluid across diagnoses and its importance as a developmental medium. Taken together, our findings establish the NHP as a potentially ideal translational model for the study of human amniotic fluid biology.

The complexity of the amniotic fluid proteome is further amplified by the evolution of the proteome over the course of gestation in healthy pregnancies, which has to date only been sparsely reported and limited to cross-sectional samples^12,18^. This study utilized amniotic fluid samples collected longitudinally from rhesus macaques at ~ G85, G110, and G135 (approximately equivalent to human gestational weeks 20, 26, and 33) and relative gestational age-matched human amniotic fluid samples collected cross-sectionally. These data reported herein are consistent with previous reports that utilized methods with less sensitivity or reduced gestational time points^12,18^. When comparing the recent report of Bhatti et al.^18^, of 320 differentially expressed human amniotic fluid proteins to the 440 differentially expressed human amniotic fluid proteins reported in this study, we found that approximately 50% of proteins were identified in both datasets, with half of those overlapping proteins varying consistently in both studies. There were differences between Bhatti et al.^18^ and our study in the timing of sample collection (17–20 weeks and term, versus ~ 20, 26, and 32 weeks) and proteomics methods used (protein array versus mass spectroscopy-based), however both studies indicate a reorganization of amniotic fluid composition with the progression of fetal development. The proteomic methods used herein have several strengths that improve proteome data quality. Isobaric labeling of tryptic peptides allows for a combination of both protein identification and quantification with high sensitivity, there is little missing data, and synchronous precursor selection mass spectroscopy (SPS-MS3) acquisition improves the dynamic range of the reporter ion measurements (detailed methods available in Supplemental Material).

Importantly, the evolution of the human amniotic fluid proteome across gestation is replicated in the NHP model with longitudinally-collected samples, suggesting that these changes in amniotic fluid protein composition across gestation may have shared biological relevance among primate species. Only in the early third-trimester did we observe differences in the trends of some human and NHP amniotic fluid proteins (Fig. 6). Specifically, rhesus proteins expressed by gastrointestinal tissues appeared to plateau while human gastrointestinal proteins continued to change. One possible explanation for this difference may be an earlier ability of the rhesus macaque infant to digest solid foods when compared to human infants (1–2 weeks postnatally versus 4–6 months)^34–36^. These results also suggest that the rhesus model may be used for the study of amniotic fluid biology, and to discover and validate biomarkers of pregnancy complications relevant to human health. For example, in a previous study of amniotic fluid from spontaneous preterm birth associated with infection/inflammation, lipocalin-1 (LCN1) was among several biomarkers identified and was decreased in cases versus controls^37^. In this study, we observed a ~ 26-fold increase in LCN1 in human amniotic fluid collected at G85 to G135 rhesus equivalent windows, in support of an important role of this protein in normal fetal development. A separate study of amniotic fluid proteins using mass spectrometry and validation by ELISA identified lumican (LUM), apolipoprotein (APOA4), and kininogen-1 (KNG1) as biomarkers of preterm birth^38^. Lumican is a major proteoglycan expressed in the cornea and skin, as well as the placenta, where it is hypothesized to play a role in preeclampsia^39^. In our study, LUM was the 9th most abundant protein in human amniotic fluid and the 22nd most abundant in rhesus amniotic fluid, and was downregulated in both species with increasing gestational age. The clinical significance of LUM concentration in amniotic fluid is unknown, but previous studies have observed association with preterm delivery as well as intra-amniotic inflammation and/or microbial invasion of the amniotic cavity in women with early preterm labor^38,40^.

It is possible that the diverse array of proteins, including bioactive proteins, and the large-scale alteration of composition and abundance across pregnancy is a by-product of fetal development and the maternal–fetal interaction over the course of gestation. However, the sheer complexity of the amniotic fluid protein composition and its cross-species reproducibility hint at the potential for amniotic fluid to directly influence fetal development through its rich and importantly, dynamic array of bioactive molecules. Use of GO, KEGG, and tissue enrichment analysis of the human and rhesus amniotic fluid data indicated increases and decreases in families of proteins associated with a diverse array of cellular functions and tissues. Specifically, we observed an increasing abundance of proteins expressed by respiratory tissues (e.g. lung and salivary gland) and decreasing abundance of proteins overrepresented in placental and gastrointestinal tissues. The increasing presence of proteins associated with lung, such as surfactant proteins SFTPB, C, and D, biologically align with preparation of the fetal lung for breathing and gas exchange after birth through production and regulation of surfactant. In addition to its role in surfactant homeostasis, SFTPD is a collectin that may function in amniotic fluid to prevent intraamniotic infection^41^ through innate immune defense. Similarly PIP, which is involved in regulation of water transport in lung airways, salivary glands, sweat glands, and apocrine glands including the mammary gland^42^, also increases in abundance across gestation. Interestingly, PIP is an aspartyl protease that cleaves fibronectin, thus its increasing abundance in amniotic fluid throughout gestation may have a relationship to the biological processes leading to delivery, and also may play a role in immune regulation^43^. Although we anticipated an increase of fetal gastrointestinal proteins with advancing gestational age to prepare for milk digestion after delivery, we observed an increase in esophageal proteins that may serve that purpose.

Further investigation is needed to better understand these observed profiles across gestation. Interestingly, 8 of the top 10 most upregulated proteins in human amniotic fluid (LCN1, LYZ, PRR4, ZG16B, LTF, PIP, S100A8, and S100SA9), have been described as biomarkers for tear fluid and proteins involved in ocular diseases^44^. The tear fluid proteome is enriched for proteins involved in immune function, which may also explain the biological abundance of these proteins in amniotic fluid^45^. Interestingly, several antimicrobial proteins differentially expressed in nasal mucosa from patients with chronic rhinosinusitis (e.g. BPIFA1, BPIFB1, BPIFB2, LTF, LYZ, S100A8, S100A9)^46^ were upregulated in amniotic fluid with increasing gestational age. Amongst those, (BPIFA1, BPIFB1, and BPIFB2) function as antimicrobial proteins^46^ and are related to immune and inflammatory processes, which may help prime the fetus for the process of labor and the postnatal environment^18,28,47^. The biological relevance of amniotic fluid proteome composition and dynamics and the potential role(s) of amniotic fluid in fetal development remain to be defined. However, continued efforts to characterize the composition of amniotic fluid over the course of gestation, by leveraging the NHP model and longitudinal sampling, may provide additional insight.

### Strengths and limitations

This study leverages the translational rhesus macaque model to address the critical knowledge gaps in our understanding of normal amniotic fluid proteome composition across gestation. Strengths of this model include similar physiology, genetic, and developmental properties with humans and the ability to perform serial biological sampling and experimental manipulation that are not feasible or ethical in humans.

We employed isobaric labeling quantitative proteomics because of the high fraction of identifiable proteins that can be quantified with little missing data. Increased multiplexing capacity and advanced data acquisition methods that improve reporter ion signal quality are also isobaric labeling strengths. We quantified 1269 proteins in rhesus amniotic fluid (21 samples) and 1381 in human amniotic fluid (24 samples) with 1090 uniquely mapping orthologs. The coefficient of variation for the human and rhesus proteomes at each gestational age ranged between 24 and 30%, lower than inter- and intra-subject variability reported in other biofluids^48,49^, despite differences in the underlying etiology for performing amniocenteses, and we observed striking similarity across species in protein abundance and dynamics.

There are several differences between the recent cross-sectional report of the human amniotic fluid proteome by Bhatti et al.^18^, and the data presented here: differences in amniotic fluid gestational age time-points and methodological differences in proteomics platforms that limit comparison between the two reports. While our methods detected ~ 1300 amniotic fluid proteins after albumin depletion, larger numbers of reported human amniotic fluid proteins have been reported after top 14 protein depletion^26^, and future work may allow for a deeper comparison of the amniotic fluid proteome across gestation, as well as between the human and rhesus models. The size of our NHP cohort was relatively small (n = 7), however this limitation is balanced by our longitudinal, repeated-measures design, which minimizes inter-sample variability. Due to the small sample size, we were not able to stratify our analysis by fetal sex, which should be a focus of future investigations on amniotic fluid composition. Additional strengths of this study are that all rhesus amniotic fluid samples were collected by a single board-certified Maternal Fetal Medicine specialist under ultrasound guidance, and that NHPs are selected with similar age, size, and housing conditions, which eliminates many of the confounders of human cross-sectional study designs.

## Conclusion

This is the first study to perform parallel analyses of rhesus and human amniotic fluid proteomes across gestation. Overall, we demonstrated high homology across these two species in amniotic fluid proteome composition and dynamics with gestational age, with large-scale evolution of protein identity and abundance. Importantly, our study suggests that the rhesus macaque model can be used for future investigations into the biological role of the amniotic fluid proteome in normal pregnancy, fetal development, and may also be relevant in developing biomarkers of obstetrical or fetal complications.

## Materials and methods

### Experimental design

All methods were carried out in accordance with relevant guidelines and regulations. Protocols were all approved by the Oregon Health & Science University (OHSU) Institutional Review Board (IRB) for human amniotic fluid (#20623), and the Oregon National Primate Research Center (ONPRC) Institutional Animal Care and Use Committee for rhesus amniotic fluid (IP0001389). Non-human primate methods are reported in accordance with the ARRIVE guidelines (https://arriveguidelines.org)^50^. This study used indoor-housed, reproductive age female rhesus macaques (n = 7) maintained on a standard chow diet (TestDiet, St. Louis, Missouri). Human amniotic fluid samples were obtained from the OHSU Knight Diagnostic Laboratory where informed consent was previously achieved to utilize the samples for research purposes.

### Non-human primate amniotic fluid collection

Ultrasound-guided amniocenteses were all performed per the standard human clinical protocol by a single board-certified Maternal Fetal Medicine specialist (J.O.L). All animals (n = 7) underwent ultrasound-guided amniotic fluid sampling on or near gestational days 85 (G85), G110, and G135 (term is ~ 168 days) (Fig. 1).

Animals were sedated by intramuscular administration of 10 mg/kg ketamine (Covetrus®) prior to intubation and maintained on 1.5% isoflurane gas with continuous monitoring of vital signs. Animals were in a dorsal recumbent position, and image-directed pulsed and color Doppler equipment with a 5- to 9-MHz sector probe (GE Voluson 730) was used to measure the amniotic fluid index, placental location, and confirmation of complete chorion and amnion fusion prior to sterile preparation of the skin. A 22-gauge needle was introduced transcutaneously under direct ultrasound guidance into the amniotic cavity, avoiding the placenta, and a 10 mL fluid sample aspirated, centrifuged at 1500×*g* for 10 min, aliquoted, and stored at − 80 °C.

### Human amniotic fluid collection

Standard clinical amniocentesis was performed by a board-certified OHSU Maternal Fetal Medicine physician. Patients were unsedated and in a supine position. 2% chlorhexidine gluconate solution was used to sterilize the abdomen and under direct ultrasound guidance, a 20-gauge needle was introduced transcutaneously into the amniotic cavity while avoiding the placenta. A total of 30 mL of fluid was aspirated, and sent to OHSU Knight Diagnostic Laboratory, where the samples were centrifuged at 2000×*g* for 10 min to remove amniocytes. Samples were aliquoted and stored at − 80 °C.

### Amniotic fluid proteome

#### Experimental design

The rhesus macaque amniotic fluid sample group comprised of 7 animals longitudinally sampled at 3 gestational ages (in days): G85, G110, and G135 (21 samples total). These gestational time points were matched by percent completed gestation with 7 human samples at each time point (21 samples total). 3 additional human samples at the G110-equivalent human gestational age were included for a total of 24 human samples. The comparable human gestational age time point for G85 is approximately 20 weeks (sample range 19 5/7 weeks to 20 4/7 weeks), for G110 approximately 25.5 weeks (sample range 24 1/7 to 26 2/7 weeks), and for G135, approximately 31.5 weeks (sample range 30 3/7 to 31 3/7 weeks) (Fig. 1). Human fetal indications for the amniocenteses are as indicated (Supplementary Table 1), with samples selected from pregnancies with the least pathologic fetal indications as possible (e.g. genetic testing with a normal fetal anatomic survey).

#### LC–MS proteomics analysis of human and rhesus amniotic fluid

After sample collection, amniotic fluid samples were prepared for proteomics analysis (for detailed methods, see Supplemental Material). Samples were depleted of albumin using an albumin depletion spin cartridge (ThermoFisher Scientific, Waltham, MA). Samples were reduced and alkylated and digested with trypsin using the EasyPep Mini digestion kit (ThermoFisher Scientific, Waltham, MA). Amniotic fluid peptides were labeled with tandem mass tag (TMTpro) 18-plex reagent kits (ThermoFisher Scientific, Waltham, MA). TMTpro labeled peptides were combined and analyzed by LC–MS on a Dionex Ultimate HPLC operating in 2-D mode coupled to an Orbitrap Fusion Tribrid mass spectrometer (Thermo Fisher Scientific, Waltham, MA) using the SPS MS3 scan mode for TMT quantitation.

Data was processed using the PAW pipeline^51^ using the Comet search engine (version 2016.03)^52^ in parallel searches of Uniprot proteomes UP000006718 (Macaca mulatta, taxon ID 9544) with canonical FASTA sequences (21,880 proteins plus common contaminants and sequence-reversed decoys) and UP000005640 (Homo sapiens, taxon ID 9606, 20,607 proteins) for identification and quantifications of proteins from each species. Statistical analysis was performed using the Bioconductor package edgeR^53^ within Jupyter notebooks.

##### Demographics

The clinical characteristics of subjects included in this study were summarized in Table 1 and Supplementary Table 1.

##### Comparative abundance across species

For each species, Internal Reference Scaling (IRS) was performed separately as previously described^54^. The coefficient of variation (CV) for each species and gestational window was calculated by dividing the standard deviation of each protein by the mean, and is reported as the median percent across all proteins. The data was then loaded into an *edgeR* DGEList object and normalized using TMM^53^, library scaling, and log transformation. The logcpm matrix for each species was used for principal component analysis using the stats package in R (version 4.1.3). The rhesus and human logcpm matrices were merged based on ortholog matching to generate a scatterplot of mean protein abundance in rhesus versus human using *ggplot2* and a heatmap of log intensity for all samples and orthologs using *ComplexHeatmap*.

##### Differential abundance with gestational age

For each species, we fit separate linear models for each protein using *limma* with gestational age in days in the design variable followed by *eBayes* moderation of standard errors. For rhesus, we also added a blocking argument and correlation value to account for within-subject variance. The resulting coefficients (logFC) from the linear models therefore describe the change in protein abundance for each day of gestation. The “AveExpr” represents the average log intensity for all samples per protein. We considered an FDR adjusted p-value < 0.05 as significant. We used the *VennDiagram* package to visualize the overlap of gestational-age associated proteins between species and to calculate enrichment of overlap using the hypergeometric test.

##### Functional enrichment analysis

We used the *clusterProfiler* and *enrichplot* packages in R version 4.2.2 to test for and visualize enrichment of GO and KEGG pathways. Proteins differentially expressed with gestational age (FDR p < 0.05) were mapped to human Entrez gene identifiers for each dataset (rhesus and human). Enrichment analyses were performed using the *compareCluster* function separately with *enrichGO* and *enrichKEGG* with the default background gene list of all genes in the database. Redundant terms were simplified using a correlation cutoff of 0.7. An adjusted p-value < 0.05 was considered significant.

##### Tissue-specific expression enrichment

To determine potential tissue sources of proteins differentially expressed with gestational age, we used the *TissueEnrich* R package^55^. We defined tissue enrichment based on RNA-seq data across 35 human tissues in the Human Protein Atlas dataset^17^. Genes with an expression level greater than 1 (TPM or FPKM) that also have at least five-fold higher expression levels compared to all other tissues were classified as enriched for a particular tissue. This package uses the hypergeometric test to calculate tissue-specific gene enrichment where, N is the total number of genes, K is the total number of tissue-specific genes for a tissue, n is the number of genes in the input gene set, and k is the number of tissue-specific genes in the input gene set. An adjusted p-value of 0.05 was considered significant to identify enriched tissues. To visualize the overall longitudinal pattern of expression in a given enriched tissue, we normalized expression for each protein by subtracting the mean and dividing by the standard deviation calculated from the reference group (~ G85 rhesus equivalent). We then take the mean across proteins enriched for a given tissue to obtain tissue-specific Z-scores and plot longitudinally relative to rhesus GA equivalent.

##### Comparison with previous studies

We compared gestational age associated protein changes in human amniotic fluid from this study with results from a previous cross-sectional study measured using a high-throughput aptamer-based platform^18^. We used the *VennDiagram* package to visualize the overlap of gestational-age-associated proteins between studies and to calculate enrichment of overlap using the hypergeometric test. For proteins identified in both studies, we compared the effect sizes using a scatterplot and Pearson correlation between logFC with gestational age.

### Supplementary Information

Supplementary Information.

Supplementary Figure 1.

Supplementary Figure 2.

Supplementary Figure 3.

Supplementary Table 1.

Supplementary Table 2.

Supplementary Table 3.

Supplementary Table 4.

Supplementary Table 5.

Supplementary Table 6.

## Data Availability

The mass spectrometry proteomics data have been deposited to the ProteomeXchange Consortium (http://proteomecentral.proteomexchange.org) via the PRIDE partner repository^56^ with the dataset identifier PXD043519.
