# Author Correction: The amniotic fluid proteome changes across gestation in humans and rhesus macaques

**DOI:** 10.1038/s41598-023-44855-4

**Published:** 2023-10-17

**Authors:** Lyndsey E. Shorey-Kendrick, B. Adam Crosland, Eliot R. Spindel, Cindy T. McEvoy, Phillip A. Wilmarth, Ashok P. Reddy, Keith D. Zientek, Victoria H. J. Roberts, Rahul J. D’Mello, Kimberly S. Ryan, Amy F. Olyaei, Olivia L. Hagen, Matthew G. Drake, Owen J. T. McCarty, Brian P. Scottoline, Jamie O. Lo

**Affiliations:** 1 Division of Neuroscience, Oregon National Primate Research Center, Oregon Health & Science University, Beaverton, OR USA; 2 Division of Maternal-Fetal Medicine, Department of Obstetrics and Gynecology, Oregon Health & Science University, Portland, OR 97239 USA; 3 Division of Neonatology. Department of Pediatrics, Oregon Health & Science University, Portland, OR USA; 4 Proteomics Shared Resources, Oregon Health & Science University, Portland, OR USA; 5 Division of Reproductive & Developmental Sciences, Oregon National Primate Research Center, Oregon Health & Science University, Beaverton, OR USA; 6 Division of Pulmonary and Critical Care Medicine, Oregon Health & Science University, Portland, OR USA; 7 Department of Biomedical Engineering, Oregon Health & Science University, Portland, OR USA

Correction to: *Scientific Reports* 10.1038/s41598-023-44125-3, published online 09 October 2023

The original version of this Article contained an error in the Author Information section.

“These authors contributed equally: Brian P. Scottoline and Jamie O. Lo.”

now reads:

“These authors contributed equally: Lyndsey E. Shorey-Kendrick and B. Adam Crosland.”

“These authors jointly supervised this work: Brian P. Scottoline and Jamie O. Lo.”

The original Article has been corrected.

