# Supplementary material for: The amniotic fluid proteome changes across gestation in humans and rhesus macaques
Source: Sci Rep. 2023 Oct 9;13:17039. doi: 10.1038/s41598-023-44125-3 (PMC10562452; doi:10.1038/s41598-023-44125-3)
Supplement: Supplementary file 1 — Supplementary Information. [file 41598_2023_44125_MOESM1_ESM.docx]

**Supplemental Methods: Detailed Proteomic Methods**

***Experimental Design***

The rhesus macaque amniotic fluid (AF) sample group comprised of 7 animals longitudinally sampled at 3 gestational ages (in days): G85, G110, and G135 (21 samples total). These gestational time points were matched by percent completed gestation with 7 human samples at each time point (21 samples total). 3 additional human samples at the G110-equivalent human gestational age were included for a total of 24 human samples. Human samples were selected from pregnancies with the least pathologic fetal indications as possible (e.g., genetic testing with a normal fetal anatomic survey).

***Digestion of amniotic fluid for proteomics analysis***

After sample collection, AF samples were stored in polypropylene centrifuge tubes at -80°C until digestion. To determine the protein concentration, a Pierce BCA protein assay (ThermoFisher Scientific, Waltham, MA, Part# 23225) was performed on water-diluted AF samples.

Rhesus and human serum albumin were removed from the AF samples using a Human Serum Albumin Affinity Removal System spin cartridge (Agilent, Santa Clara, CA, Part#5188-5334). To prepare the samples for albumin depletion, a 400 µL aliquot of AF was transferred to a 1.5mL Protein LoBind centrifuge tube (VWR, Radnor, PA, Part# 80077-232) and centrifuged at 14,000 xg for 10 min. The supernatant was filtered through a 0.22 µm Ultrafree-MC PVDF spin filter (Millipore, Burlington, MA, Part# UFC30GVNB), to remove cells and particulates, and the samples were centrifuged until all the amniotic fluid had passed through the filter. The 0.22 µm filtrate was transferred to an Amicon Ultracel-3K 3,000 Dalton (Da) cutoff centrifugal filter (Millipore, Burlington, MA, Part# UFC500396) for concentration and buffer exchange with multiple centrifugations at 14,000 xg to concentrate the sample to ~100 µL volume followed by dilution in Depletion Column Buffer A (Agilent, Santa Clara, CA, Part# 5185-5987). The buffer-exchanged retentate was collected by inverting the 3,000 Da centrifugal filter into a clean 2.0 mL centrifuge tube and centrifuged at 3,000 xg for 3 min. The volume was measured, and additional Depletion Column Buffer A was added to bring the final volume to 200 µL.

The depletion cartridge was equilibrated with Depletion Column Buffer A and the 200 µL AF sample was added to the top of the cartridge. The cartridge was centrifuged at 100 xg for 1.5 min and the flow through was collected, followed by two column washes with Depletion Column Buffer A. The depletion spin cartridge was cleaned by eluting the bound human serum albumin with 400 µL of Depletion Column Buffer B (Agilent, Santa Clara, CA, Part# 5185-5988), and the elution fluid was set aside. The three albumin-depleted flow through fractions were combined and concentrated in a new 3,000 Da centrifugal spin filter. Samples were added in 400 µL increments to the centrifugal filter which was centrifuged at 14,000 xg for 10 min between additions. The concentrated sample retentate was collected by inverting the 3,000 Dalton centrifugal filter into a clean 2.0 mL centrifuge tube and centrifuged at 3,000 xg for 3 min. The volume was measured, and a BCA protein assay was performed to determine the post-depletion protein recovery. The average amount of protein in the serum albumin depleted samples was 422 µg with an average recovery of 36.1%.

The serum albumin-depleted AF sample proteins were subjected to proteolytic digestion using an EasyPep Mini Digestion kit (ThermoFisher Scientific, Waltham, MA) as follows: The concentrated AF samples were dried using a Speedvac centrifugal vacuum. The resulting pellet was resuspended in 100 µL of EasyPep Mini lysis buffer and incubated at 50°C for 15 min. The samples were reduced, and the cysteines alkylated by adding 50 µL each of the EasyPep Mini Reduction Solution and the EasyPep Mini Alkylation Solution and incubating the resultant mixture at 95°C for 10 min. Trypsin/Lys-C was added, and the samples were incubated with shaking at 37°C for 3 hours. The digestion was halted with 50 µL of Digestion Stop Solution and the samples were gently mixed for 10 sec.

Digested samples were cleaned using an EasyPep Mini Peptide Clean-up column as follows: The digest solution was transferred to a dry Clean-up column and centrifuged at 1,500 xg for 2 min. The column was washed once with 300 µL of Wash Solution A and twice with 300 µL of Wash Solution B with the buffer being removed by centrifugation at 1,500 xg for 2 min in between washes. Peptides were eluted from the Peptide Clean-up column using 300 µL of the Elution Solution followed by centrifugation at 1,500 xg for 2 min to collect the peptide-containing eluent. The eluent was Speedvac centrifugal vacuum dried, and the peptide samples reconstituted in 100µL of 0.1% formic acid in dionized water and peptide concentrations determined using a Pierce Quantitative Colorimetric Peptide Assay (ThermoFisher Scientific, Cat # 23275). Aliquots equaling 2.1 µg of peptides were removed from all samples and pooled together to form the internal reference standard samples (common pool) used for internal reference scaling^1^ (IRS) data analysis. For the Tandem mass tag (TMTpro) labeling step, 14 µg of the peptide from each AF sample and the common pool samples were then dried by vacuum centrifugation. Dried peptides were reconstituted by adding 100 µL of 100mM Triethanolamine buffer (TEAB) and shaking at 37°C for 15 min.

***TMTpro labeling and normalized mixing***

Three tandem mass tag (TMTpro) 18-plex reagent kits (ThermoFisher Scientific, Cat # 90309) were used to label the digested AF peptide samples and pooled standards. Twelve µL of anhydrous acetonitrile was added to 100 µg portions of TMTpro 18-plex reagents, the peptide samples in 20 µL of 100mM TEAB were immediately transferred into the dissolved TMTpro reagents, and labeling was performed by shaking at room temp for 1 hr. After the incubation, 2 µL of each labeled peptide was combined, and 2 µL of 5% hydroxylamine was added. Samples were incubated at room temp for 15 min, then dried by vacuum centrifugation. The remaining 30 µL of each labeled sample was frozen at -80°C without hydroxylamine addition, in case relabeling was required.

The 2 µL of each combined TMTpro labeled sample was then dissolved in 20 µL of 5% formic acid and 2 µg of peptides analyzed by a single 140 min LC-MS/MS method using an Orbitrap Fusion Mass Spectrometer (HPLC system and mass spectrometer described below). These single LC runs were performed to volumetrically adjust each sample to provide similar total reporter ion intensities for each labeled sample in the final combined mixture for the 2D-LC/MS analysis. The remaining 30 µL portion of each TMTpro labeled sample was then thawed and aliquots were removed and adjusted by the calculated normalization factors that would produce 50 µg of total peptides from all samples in each TMTplex experiment. To quench the labeling reaction, 5% hydroxylamine was added to bring the total hydroxylamine concentration to 0.5% followed by incubation for 15 min at room temperature. The remaining labeled peptides not used for the 2D-LC/MS were stored at -80°C in case a second run was necessary.

***Two-dimensional liquid chromatography/mass spectrometry (2D-LC/MS) analysis***

The multiplexed samples and common pool samples were dissolved in 10 mM ammonium formate, pH 9 buffer and injected onto a NanoEase 5 µm XBridge BEH130 C18 300 µm x 50 mm column (Waters Corporation, Milford, MA) at 3 µL/min in a mobile phase containing 10 mM ammonium formate (pH 9). Peptides from AF were eluted by sequential injection of 20 µL volumes of 17, 20, 21, 22, 23, 24, 25, 26, 27, 28, 29, 30, 31, 32, 33, 34, 35, 40, 50, 90% acetonitrile (ACN) (20 fractions).

Eluted peptides were diluted at a 3-way union with mobile phase containing 0.1% formic acid at a 24 µL/min flow rate and delivered to an Acclaim PepMap 100 µm x 2 cm NanoViper C18, 5 µm trap (Thermo Fisher Scientific) on a switching valve. After 10 min of loading, the trap column was switched in-line to a PepMap RSLC C18, 2 µm, 75 µm x 25 cm EasySpray column (ThermoFisher Scientific). TMTpro 18-plex labeled peptides from AF were then separated at low pH in the second dimension using a 7.5–30% ACN gradient over 100 min in the mobile phase containing 0.1% formic acid at a 300 nL/min flow rate.

Tandem mass spectrometry data was collected using an Orbitrap Fusion Tribrid instrument (Thermo Scientific, San Jose, CA) configured with an EasySpray NanoSource. Survey scans were performed in the Orbitrap mass analyzer (400-1600 m/z range, resolution = 120,000, AGC = 400,000, MIT = 50 ms), with internal mass calibration enabled, and data-dependent MS2 scans using dynamic exclusion (window 10 ppm, duration 60s) performed in the linear ion trap using collision-induced dissociation (NCE = 30, Rapid mode, 450-1400 m/z range, AGC = 10,000, MIT = 50 ms). Reporter ion detection was performed in the Orbitrap mass analyzer (resolution = 50,000) using MS3 scans following synchronous precursor isolation of the top 10 ions in the linear ion trap, and higher-energy collisional dissociation (NCE = 55; 110-500 m/z range, AGC = 125,000, MIT = 120 ms) in the ion-routing multipole.

***Data analysis***

FASTA files for each species were downloaded from [www.UniProt.org](http://www.UniProt.org) in October 2022. Proteome UP000006718 (Macaca mulatta, taxon ID 9544) canonical (one protein per gene) FASTA had 21,880 protein sequences. Common contaminants (175 sequences) were added, and sequence-reversed entries were concatenated for a final FASTA file of 44,110 sequences. UP000005640 (Homo sapiens, taxon ID 9606) canonical FASTA had 20,607 protein sequences and was augmented with common contaminants and decoy sequences for a final FASTA file with 41,564 sequences.

The 60 binary instrument files (20 fractions in each of the three plexes) were processed with the PAW pipeline^1^ (https://github.com/pwilmart/PAW_pipeline). Binary files were converted to text files using MSConvert^2^. Python scripts extracted TMTpro reporter ion peak heights and fragment ion spectra in MS2 format^3^. The Comet search engine (version 2016.03)^4^ was used: 1.25 Da monoisotopic peptide mass tolerance, 1.0005 Da monoisotopic fragment ion tolerance, semi tryptic cleavage with up to two missed cleavages, variable oxidation of methionine and proline residues, static alkylation of cysteines, and static modifications for TMTpro labels (at peptide N-termini and at lysine residues).

Two parallel analyses were done for searches using the rhesus FASTA file and for searches using the human FASTA file. Samples from both species were present in each of the three plexes and aliquots of peptide digests from all 45 samples (both species) were combined for the internal reference channels. The methods described below were done independently for each species.

Top-scoring peptide spectrum matches (PSMs) were filtered to a 1% false discovery rate (FDR) using interactive delta-mass and conditional Peptide-prophet-like linear discriminant function scores^5^. Incorrect delta-mass and score histogram distributions were estimated using the target/decoy method^7^. The filtered PSMs were assembled into protein lists using basic and extended parsimony principles and required two distinct peptides per protein. The final list of identified proteins, protein groups, and protein families were used to define unique and shared peptides for quantitative use. Total (summed) reporter ion intensities were computed from the PSMs associated with all unique peptides for each protein.

The quantitative data for each plex was put on a common intensity scale using the internal reference scaling method described in Plubell et al. 2017^6^. The intensities of the duplicate pooled standard channels in each plex were used to compute scaling factors to correct for the pseudo-random MS2/MS3 scan selection process and make the pooled standard channel averages in each plex identical. Those scaling factors were applied to all channels containing the biological samples in each plex. IRS was done in parallel for each species using their respective FASTA search results. IRS, when using results from the rhesus FASTA search, puts all the rhesus sample channels across the three plexes on a common intensity scale (human sample channels were ignored in rhesus FASTA search results). IRS with the human FASTA search results similarly puts all human sample channels on a common intensity scale (rhesus samples were ignored in human FASTA results).

The protein intensity values for each biological sample in each biological condition were compared for differential protein expression using the Bioconductor package edgeR^7^ within Jupyter notebooks for each species. Basic differential analyses involved using the trimmed mean of M-values^8^ compositional data normalization and the exact pairwise testing. Linear modeling options were also used to do ANOVA testing. Result tables contained typical proteomics summaries, reporter ion intensities, and statistical testing results. Additional annotations from [www.UniProt.org](http://www.UniProt.org) were added (<https://github.com/pwilmart/annotations>).

The mass spectrometry proteomics data have been deposited to the ProteomeXchange Consortium (<http://proteomecentral.proteomexchange.org>) via the PRIDE partner repository^9^ with the dataset identifier PDX043519.

***Ortholog mapping***

Comparison of the rhesus amniotic fluid proteome (proteins and their relative abundances) to the human AF proteome was done as follows: Two small FASTA files were created from the final list of inferred proteins for each species. A single protein sequence was used for any inferred protein groups. The inferred rhesus AF protein sequences were compared to the inferred human AF protein sequences using NCBI BLAST called from scripts available at <https://github.com/pwilmart/PAW_BLAST>. The rhesus AF proteome was the reference state since we were more interested in how the rhesus AF proteome reflected the human AF proteome. Ortholog relationships were defined as reciprocal best matches (a common criterion). Comparing protein sequences from lists of protein present in the two AF proteomes rather than comparing all rhesus sequences to all human sequences is a key aspect of this analysis.

The resulting ortholog pair list needed some additional cleanup before relative protein abundances could be compared between species. Immunoglobulins present in amniotic fluid come from IgG and IgA complexes. There are a few hundred explicit Ig protein sequence in each species FASTA file and associations of individual Ig matches to IgG or IgA complexes is not possible. We collapsed 65 rhesus immunoglobulin proteins into a single immunoglobulin group and similarly collapsed 83 human immunoglobulins into one group. The BLAST postprocessing script is relaxed in matching query proteins (rhesus) to hit proteins (human) and more than one rhesus protein can be matched to the same human protein. BLAST match entropy scores were used to find the best ortholog pair and remove lower scoring ortholog pairs.

The 1,269 rhesus AF proteins could be mapped to 1,090 human orthologs after reducing immunoglobulins, removing non-top-scoring ortholog pairs, and any rhesus AF proteins that did not match any human AF proteins. The protein reporter ion intensity sums from these 1,090 ortholog pairs were averaged across all samples or gestational age samples for each species for further comparative analyses (scatter plot correlations, functional enrichments, etc.).

**References:**

1 Wilmarth, P. A., Riviere, M. A. & David, L. L. Techniques for accurate protein identification in shotgun proteomic studies of human, mouse, bovine, and chicken lenses. *J Ocul Biol Dis Infor* **2**, 223-234 (2009). <https://doi.org:10.1007/s12177-009-9042-6>

2 Chambers, M. C. *et al.* A cross-platform toolkit for mass spectrometry and proteomics. *Nat Biotechnol* **30**, 918-920 (2012). <https://doi.org:10.1038/nbt.2377>

3 McDonald, W. H. *et al.* MS1, MS2, and SQT-three unified, compact, and easily parsed file formats for the storage of shotgun proteomic spectra and identifications. *Rapid Commun Mass Spectrom* **18**, 2162-2168 (2004). <https://doi.org:10.1002/rcm.1603>

4 Eng, J. K., Jahan, T. A. & Hoopmann, M. R. Comet: an open-source MS/MS sequence database search tool. *Proteomics* **13**, 22-24 (2013). <https://doi.org:10.1002/pmic.201200439>

5 Keller, A., Nesvizhskii, A. I., Kolker, E. & Aebersold, R. Empirical statistical model to estimate the accuracy of peptide identifications made by MS/MS and database search. *Anal Chem* **74**, 5383-5392 (2002). <https://doi.org:10.1021/ac025747h>

6 Plubell, D. L. *et al.* Extended Multiplexing of Tandem Mass Tags (TMT) Labeling Reveals Age and High Fat Diet Specific Proteome Changes in Mouse Epididymal Adipose Tissue. *Mol Cell Proteomics* **16**, 873-890 (2017). <https://doi.org:10.1074/mcp.M116.065524>

7 Robinson, M. D., McCarthy, D. J. & Smyth, G. K. edgeR: a Bioconductor package for differential expression analysis of digital gene expression data. *Bioinformatics* **26**, 139-140 (2010). <https://doi.org:10.1093/bioinformatics/btp616>

8 Robinson, M. D. & Oshlack, A. A scaling normalization method for differential expression analysis of RNA-seq data. *Genome Biology* **11**, R25 (2010). <https://doi.org:10.1186/gb-2010-11-3-r25>

9 Perez-Riverol, Y. *et al.* The PRIDE database resources in 2022: a hub for mass spectrometry-based proteomics evidences. *Nucleic Acids Res* **50**, D543-d552 (2022). <https://doi.org:10.1093/nar/gkab1038>
