# Supplementary material for: The amniotic fluid proteome changes across gestation in humans and rhesus macaques
Source: Sci Rep. 2023 Oct 9;13:17039. doi: 10.1038/s41598-023-44125-3 (PMC10562452; doi:10.1038/s41598-023-44125-3)

Supplemental Figure 1. Enrichment of tissue-specific signatures associated with gestational age in rhesus macaques. Tissue enrichment analysis was performed in all proteins associated with gestational age in rhesus amniotic fluid, separately for up- and down-regulated proteins. For each enriched tissue, we summarized expression of all proteins over-represented in that tissue using a Z-score normalized to abundance at the earliest gestational age. The tissue-specific trajectories over gestational age are shown for upregulated (top) and downregulated (bottom) proteins for rhesus samples (left). The trajectories for these proteins in the human samples (right) are also shown.

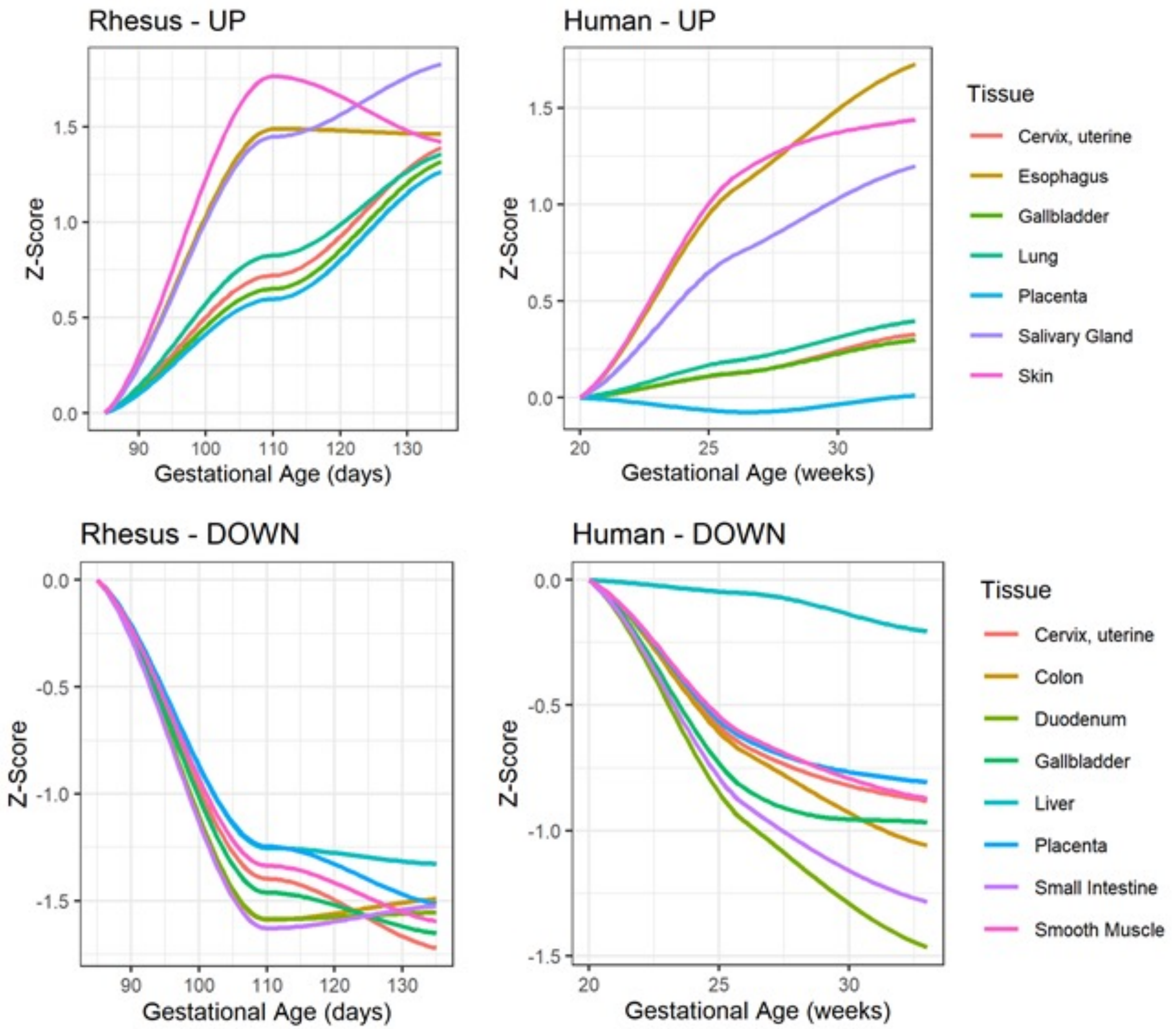

Supplement: Supplementary file 2 — Supplementary Figure 1. [file 41598_2023_44125_MOESM2_ESM.pdf]
