# Supplementary material for: The amniotic fluid proteome changes across gestation in humans and rhesus macaques
Source: Sci Rep. 2023 Oct 9;13:17039. doi: 10.1038/s41598-023-44125-3 (PMC10562452; doi:10.1038/s41598-023-44125-3)

Supplemental Figure 2. Enrichment of tissue-specific signatures associated with gestational age in humans. Tissue enrichment analysis was performed in all proteins associated with gestational age in human amniotic fluid, separately for up- and down-regulated proteins. For each enriched tissue, we summarized expression of all proteins over-represented in that tissue using a Z-score normalized to abundance at the earliest gestational age. The tissue-specific trajectories over gestational age are shown for upregulated (top) and downregulated (bottom) proteins separately for human samples (left) and for the same proteins in rhesus samples (right).

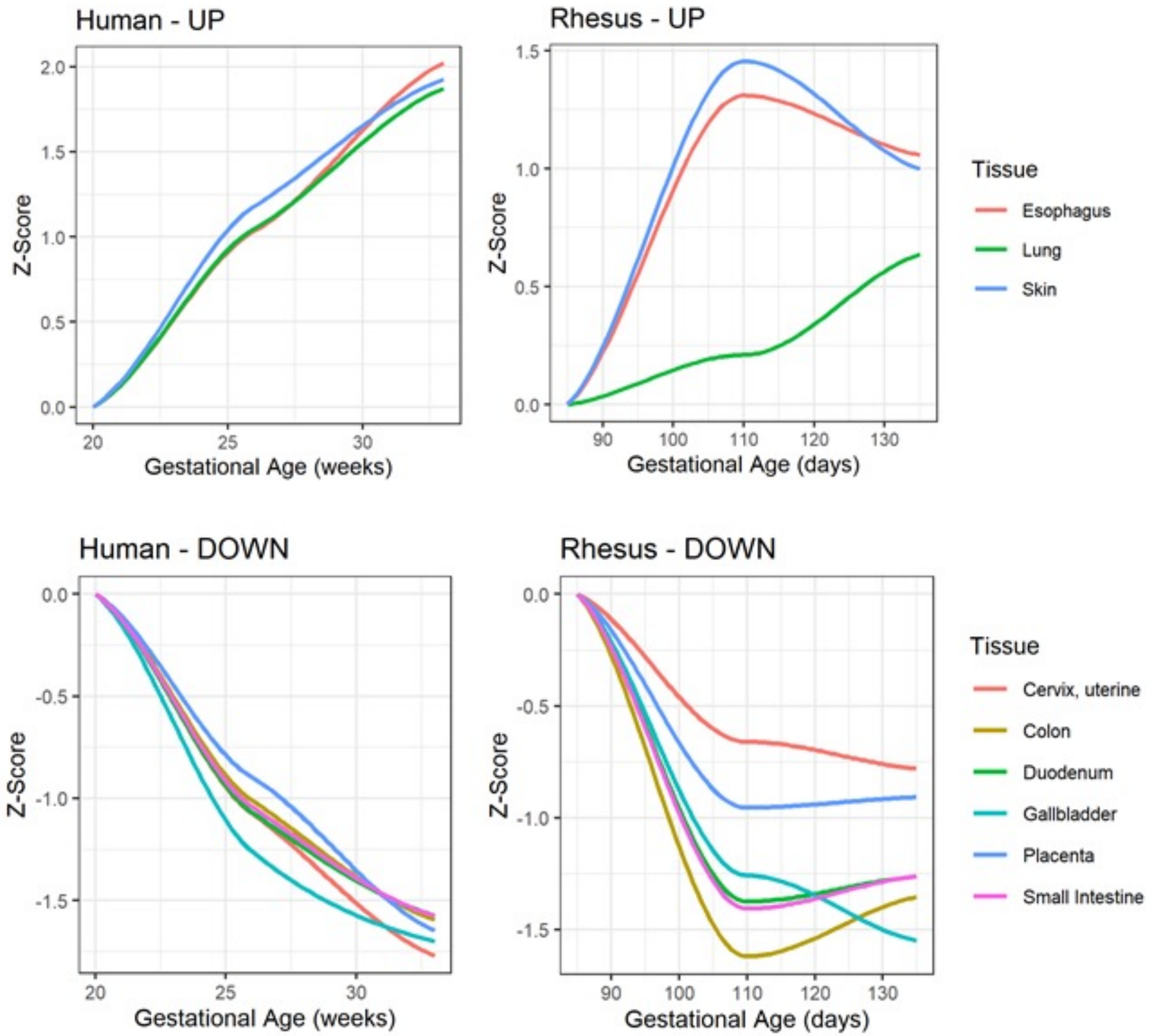

Supplement: Supplementary file 3 — Supplementary Figure 2. [file 41598_2023_44125_MOESM3_ESM.pdf]
