# Supplementary material for: The amniotic fluid proteome changes across gestation in humans and rhesus macaques
Source: Sci Rep. 2023 Oct 9;13:17039. doi: 10.1038/s41598-023-44125-3 (PMC10562452; doi:10.1038/s41598-023-44125-3)

Supplemental Figure 3. Gestational age associated protein changes are consistent with previous study. Venn diagram (A) showing the overlap of amniotic fluid proteins associated with gestational age in human samples from this study with proteins associated with gestational age in a study by **Bhatti et al.[18]. The scatterplot (B) shows the correlation of effect size in this study on the y-axis (in units of change per day of gestation) with the effect size in the Bhatti et al. study (units reflect change from mid-gestation to term).**

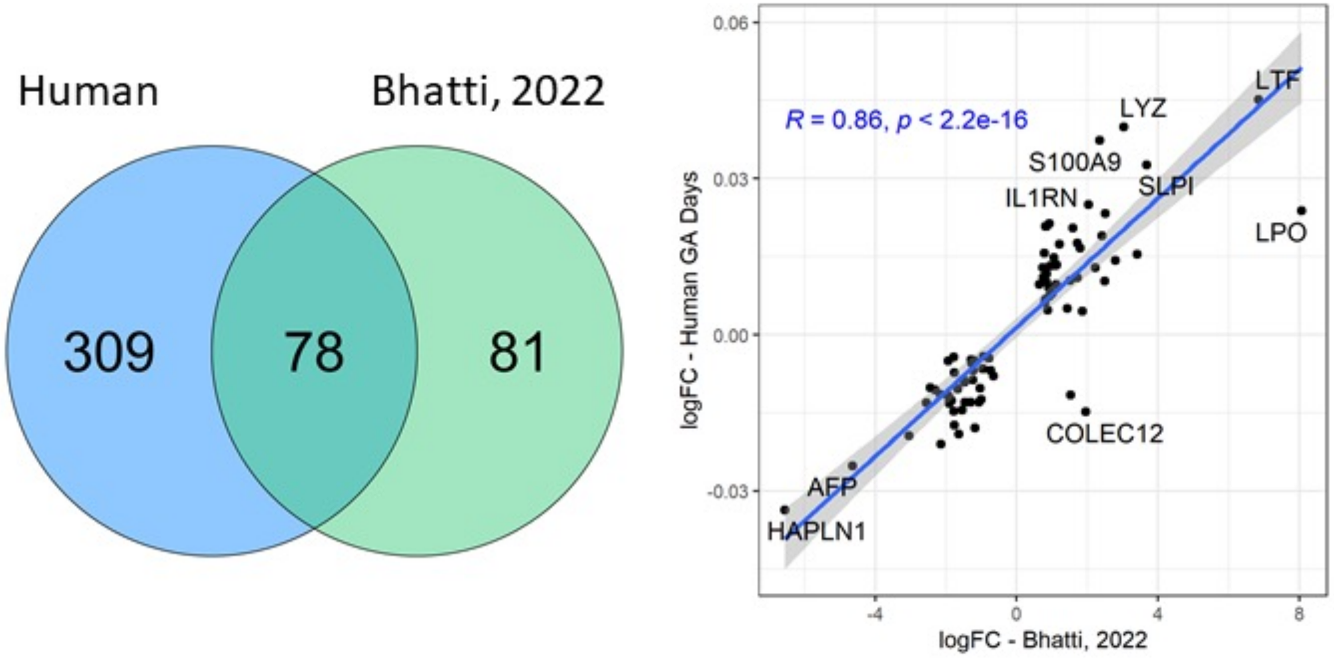

Supplement: Supplementary file 4 — Supplementary Figure 3. [file 41598_2023_44125_MOESM4_ESM.pdf]
