# Supplementary material for: The amniotic fluid proteome changes across gestation in humans and rhesus macaques
Source: Sci Rep. 2023 Oct 9;13:17039. doi: 10.1038/s41598-023-44125-3 (PMC10562452; doi:10.1038/s41598-023-44125-3)
Supplement: Supplementary file 5 — Supplementary Table 1. [file 41598_2023_44125_MOESM5_ESM.docx]

**Supplemental Table 1: Indications for Human Amniocentesis Samples**

| **Rhesus Gestational Age**  **(Days)** | **Human Gestational Age Equivalent (wks)** | **Indication for Amniocentesis** | **Diagnosis at Birth** |
| --- | --- | --- | --- |
| G85 | 19  19 1/7  19 5/7  20  20  20 2/7  20 4/7 | Genetic testing  Genetic testing  Genetic testing  Bilateral club foot  Genetic testing  Genetic testing  Unilateral antenatal urinary tract dilation grade 1 (UTDA1), echogenic intracardiac focus | Normal  Normal  Normal  Bilateral club foot  Normal  Normal  Normal |
| G110 | 24 1/7  24 3/7  24 4/7  24 6/7  25 1/7  25 3/7  25 4/7 | Unilateral club foot  Genetic testing  Possible Trisomy 21  Fetal growth restriction  Bilateral antenatal urinary tract dilation grade 2 (UTDA2)  Hypoplastic left heart syndrome  Ventricular septal defect, possible coarctation of the aorta | Unilateral club foot  Normal  Trisomy 21  Small for gestational age, otherwise normal  Normal  Hypoplastic left heart syndrome, otherwise normal  Ventricular septal defect, atrial septal defect, coarctation of the aorta, otherwise normal |
| G135 | 30 3/7  30 6/7  30 6/7  31 1/7  31 1/7  31 3/7  31 6/7 | Fetal ascites  Unilateral pleural effusion  Fetal intraventricular hemorrhage  Suspected Trisomy 21  Fetal growth restriction of twin B, dichorionic diamniotic twin pregnancy  Suspected Trisomy 21  Suspected neuronal migration defect | Ascites, otherwise normal  Normal  Intraventricular hemorrhage  Trisomy 21  Normal  Trisomy 21  Cerebral cortical malformation, congenital hypothyroidism |
